# Progress in Neurology

**Published:** 1902-08-16

**Authors:** 


### Progress in Neurology.

**Knee-jerks in Transverse Lesions of the Spinal Cord.**—Turner [1] has experimented on monkeys by making transverse section of the cord, with the following results. He finds that the condition of the knee jerks is not constant: the higher the level of the cross-section the greater the likelihood of the knee-jerks being temporarily diminished or abolished. No such difference according to level is found in trans-sections of the human spinal cord; all complete trans-sections above the lumbar enlargement lead to abolition of the knee-jerks, most probably of a permanent character; but temporary abolition, sometimes for a prolonged period, may follow incomplete anatomical trans-section, although the other co-existent phenomena point to a complete physiological lesion. In man, as experimentally in monkeys, although the knee-jerks may be abolished, some true reflex actions are permanently retained; such are the plantar and superficial anal reflexes; and, notably in monkeys, the crossed adductor jerks. We may conclude that in spinal trans-section in man, and in high trans section in monkeys, actions dependent on neuro-muscular tone are permanently or temporarily abolished, but that true reflex movements are not impaired. The variation which exists in the phenomena following lesion at different spinal levels in dogs and monkeys, the transitory effects of such lesions as regards the knee-jerks, as well as the temporary abolition of the knee-jerks in incomplete lesions in man, would preclude the general application of Bastian's theory (which ascribes the loss of the reflexes to the cutting off of cerebral and cerebellar influence); for there is nothing yet recorded to negative the view that the mechanism, which produces loss of the knee-jerks in man, or their temporary abolition in the lower animals, is not to be found in the spinal cord itself. The explanation of the discrepancy which exists between the results of trans-section in laboratory animals and man, may be explained by the greater autonomy of the spinal segments in maintaining neuro-muscular tone as we descend the animal scale.

**Reflexes.**—In connection with Turner's paper, a summary of the various clinical phenomena, by Walton,[2] is of interest. In cerebral hæmorrhage the paralysis may be at first flaccid, and the reflexes abolished. If the connection between the brain and the lumbar cord is permanently severed, the knee-jerk does not return, though the plantar reflex may do so. If the destruction of the upper cord is gradual the knee-jerk does not disappear, but becomes exaggerated. The Babinski reflex may appear at the onset of high lesion during the period of flaccidity and absence of all other reflexes. In earliest infancy, even in infants prematurely born, extensive reflex movements of the feet and toes are present; these reflex movements are gradually replaced, probably after two years, by the constant flexor reflex of adult life. These observations cannot be reconciled with the supposition of single centres, either in the cord or in the brain. The theory of shock, as maintained by Gowers, leaves much to be desired, for shock, in transverse section of the cord, should also restrain the Babinski reflex which it does not do.

**Tabes.**—Foulton [3] says that the diagnosis of early tabes from general paralysis is sometimes difficult. Paræsthesiæ about the limbs and body are often the first symptoms of tabes, occasionally difficulty in micturition. Among the earliest symptoms of general paralysis there may be found loss of knee-jerk, Argyll-Robertson pupil, lightning pains, incoordination and weakness of the bladder. If, however, the characteristic clumsiness of speech and tremor of the tongue and lower facial muscles were present, general paralysis might be diagnosed. Eighty-five per cent. of tabetic patients gave a definite history of some gastro-intestinal disturbance preceding the onset of the disease; so that it is possible that some obscure toxæmia of gastro-intestinal origin, combined with syphilis, constitute the exciting cause. According to Bramwell [4] out of 155 cases of tabes, syphilis was ascertained to have existed in 60 per cent., whilst in the remaining cases its existence was doubtful or denied. The length of time between infection and the development of tabes was less than 20 years in the vast majority, the second, third, and fourth quinquennium claiming each nearly a fourth of the victims. The commonest initial symptoms were lightning pains, diplopia and ataxy, loss of vision, derangements of urination, gastric crises, and numbness of the feet, in the above-mentioned order. Lightning pains were also the commonest symptoms in the course of the disease. Mental symptoms in the form of general paralysis developed in 16 cases, impairment of memory in five, and mental depression in four cases; but in the great majority mental activity was unimpaired even in the latter stages of the disease. In a clinical lecture on anomalous cases of tabes, Taylor[5] says that in cases of laryngeal crises ataxy is almost always a marked symptom. They consist of extreme inspiratory spasms, the result of spasmodic adduction, and in such cases there is nearly always weakness of the abductors. In view of the fact that death may suddenly result from such attacks, the question of tracheotomy as a precautionary measure should be carefully considered. The characteristic of the joint troubles is the extreme deformity of the affected joint without any pain, and in such cases there is practically no affection of the power of locomotion and no interference with co-ordination. Perforating ulcers, contrary to received opinion, do heal in very many cases, and it is not the rule that severe ataxy is present.

[1] Jour. of Nerv. and Ment. Dis., June, 1902. [2] Jour. of Nerv. and Ment. Dis., June, 1902. [3] Jour. of Nerv. and Ment. Dis., April, 1902. [4] Brain. Spring., 1902. [5] Brit Med. Jour., July 19, 1902.

